# Supplementary material for: Immediate and short-term effects of neurodynamic techniques on hamstring flexibility: A systematic review with meta-analysis
Source: PLoS One. 2025 Feb 6;20(2):e0318671. doi: 10.1371/journal.pone.0318671 (PMC11801537; doi:10.1371/journal.pone.0318671)
Supplement: S1 File — (DOCX) [file pone.0318671.s002.docx]

**SUPPORTING INFORMATION**

**S1 Table.** Search strategy for each database.

**S2 Table**. Studies excluded after full text read with the reason for exclusion

**S1 Figure**. Risk of bias.

**S2 Figure.** Sensitivity analysis of immediate effects

**S3 Figure.** Sensitivity analysis of short-term effects

**S4 Figure.** Funnel plot of immediate effects

**S5 Figure.** Funnel plot of short-term effects

**S1 table.** Search strategy for each database.

| **MEDLINE (via Pubmed)** |
| --- |
| ("athletic performance" OR performance OR "sport performance" OR flexibility OR extensibility OR ROM) AND ("nerve treatment" OR "nerve therapy" OR "neural mobilization" OR neurodynamic OR neurodynamics OR "nerve tension" OR "nerve stretch" OR "neural tension" OR "nerve mobilization" OR "neural glide" OR "neural treatment" OR "nerve gliding" OR nerve glide" OR "neural gliding" OR "nerve gliding exercises" OR “neuromobilization maneuver” OR neuromobilization OR "neurodynamic techniques") AND (RCT OR "randomized clinical trial" OR "randomized trial" OR "randomized controlled trial")  **Results: 84** |
| **Scopus** |
| **TITLE-ABS-KEY (**("athletic performance" OR performance OR "sport performance" OR flexibility OR extensibility OR ROM)) **AND  TITLE-ABS-KEY (**("nerve treatment" OR "nerve therapy" OR "neural mobilization" OR neurodynamic OR neurodynamics OR "nerve tension" OR "nerve stretch" OR "neural tension" OR "nerve mobilization" OR "neural glide" OR "neural treatment" OR "nerve gliding" OR nerve glide" OR "neural gliding" OR "nerve gliding exercises" OR “neuromobilization maneuver” OR neuromobilization OR "neurodynamic techniques")) **AND  TITLE-ABS-KEY (**(RCT OR "randomized clinical trial" OR "randomized trial" OR "randomized controlled trial"))  **Results: 65** |
| **Web Of Science (All databases)** |
| ("athletic performance" OR performance OR "sport performance" OR flexibility OR extensibility OR ROM) AND ("nerve treatment" OR "nerve therapy" OR "neural mobilization" OR neurodynamic OR neurodynamics OR "nerve tension" OR "nerve stretch" OR "neural tension" OR "nerve mobilization" OR "neural glide" OR "neural treatment" OR "nerve gliding" OR nerve glide" OR "neural gliding" OR "nerve gliding exercises" OR “neuromobilization maneuver” OR neuromobilization OR "neurodynamic techniques") AND (RCT OR "randomized clinical trial" OR "randomized trial" OR "randomized controlled trial")  **Results: 30** |
| **Cochrane Database** |
| ("athletic performance" OR performance OR "sport performance" OR flexibility OR extensibility OR ROM) AND ("nerve treatment" OR "nerve therapy" OR "neural mobilization" OR neurodynamic OR neurodynamics OR "nerve tension" OR "nerve stretch" OR "neural tension" OR "nerve mobilization" OR "neural glide" OR "neural treatment" OR "nerve gliding" OR nerve glide" OR "neural gliding" OR "nerve gliding exercises" OR “neuromobilization maneuver” OR neuromobilization OR "neurodynamic techniques") AND (RCT OR "randomized clinical trial" OR "randomized trial" OR "randomized controlled trial")  **Results: 150** |
| **Sportdiscus** |
| ("athletic performance" OR performance OR "sport performance" OR flexibility OR extensibility OR ROM) AND ("nerve treatment" OR "nerve therapy" OR "neural mobilization" OR neurodynamic OR neurodynamics OR "nerve tension" OR "nerve stretch" OR "neural tension" OR "nerve mobilization" OR "neural glide" OR "neural treatment" OR "nerve gliding" OR nerve glide" OR "neural gliding" OR "nerve gliding exercises" OR “neuromobilization maneuver” OR neuromobilization OR "neurodynamic techniques") AND (RCT OR "randomized clinical trial" OR "randomized trial" OR "randomized controlled trial")  **Results: 10** |
| **TOTAL results: 339** |

**S2 Table**. Studies excluded after full text read with the reason for exclusion.

| **Reference** | **Reason for exclusion** |
| --- | --- |
| Cihan et al, 2019 | No outcome of interest |
| Fasen et al, 2009 | No data for analysis |
| Cancela et al, 2022 | No data for analysis |
| Martins et al, 2019 | Comparison of 2 neurodynamic techniques |
| Nunes et al, 2017 | No outcome of interest |
| Pereira et al, 2020 | Comparison of 2 neurodynamic techniques |
| Pietrzak et al, 2016 | Comparison of 2 neurodynamic techniques |
| Dsouza et al, 2023 | Comparison of 2 neurodynamic techniques |
| Vedang et al, 2021 | No intervention of interest |

**S1 Figure**. Risk of bias for (A) immediate and (B) short-term effects.


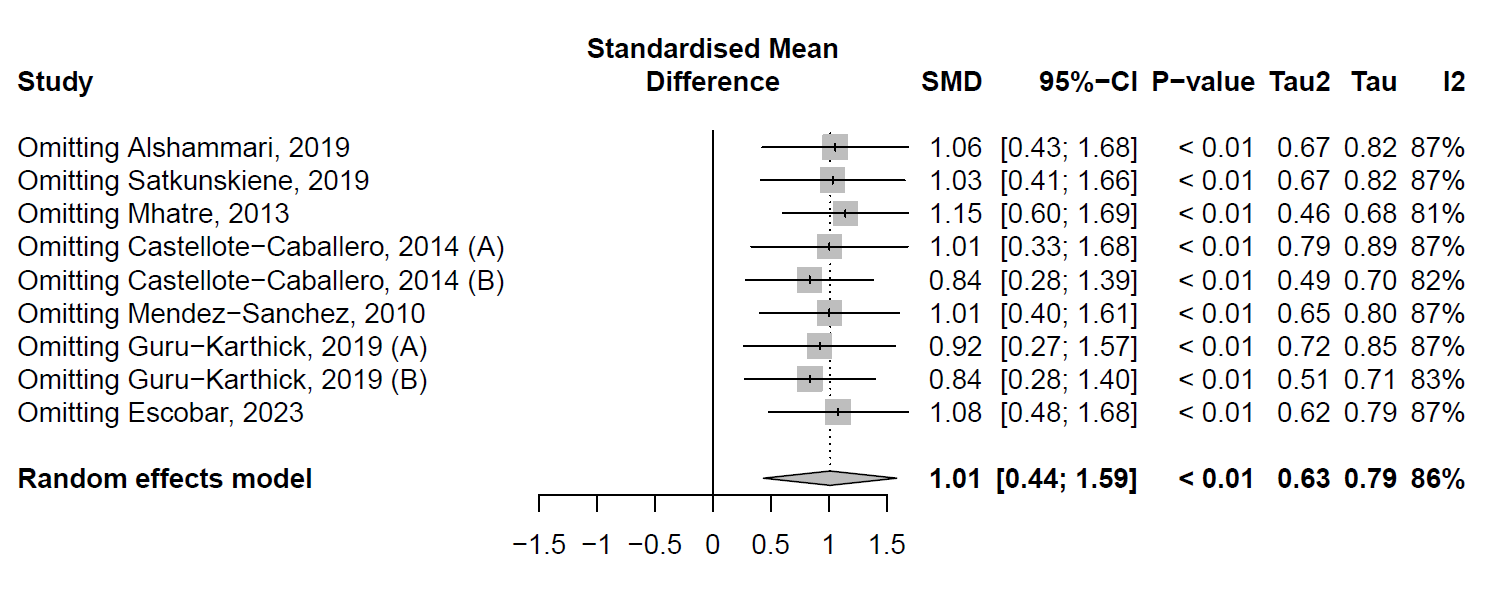


**S2 Figure.** Sensitivity analysis of immediate effects.


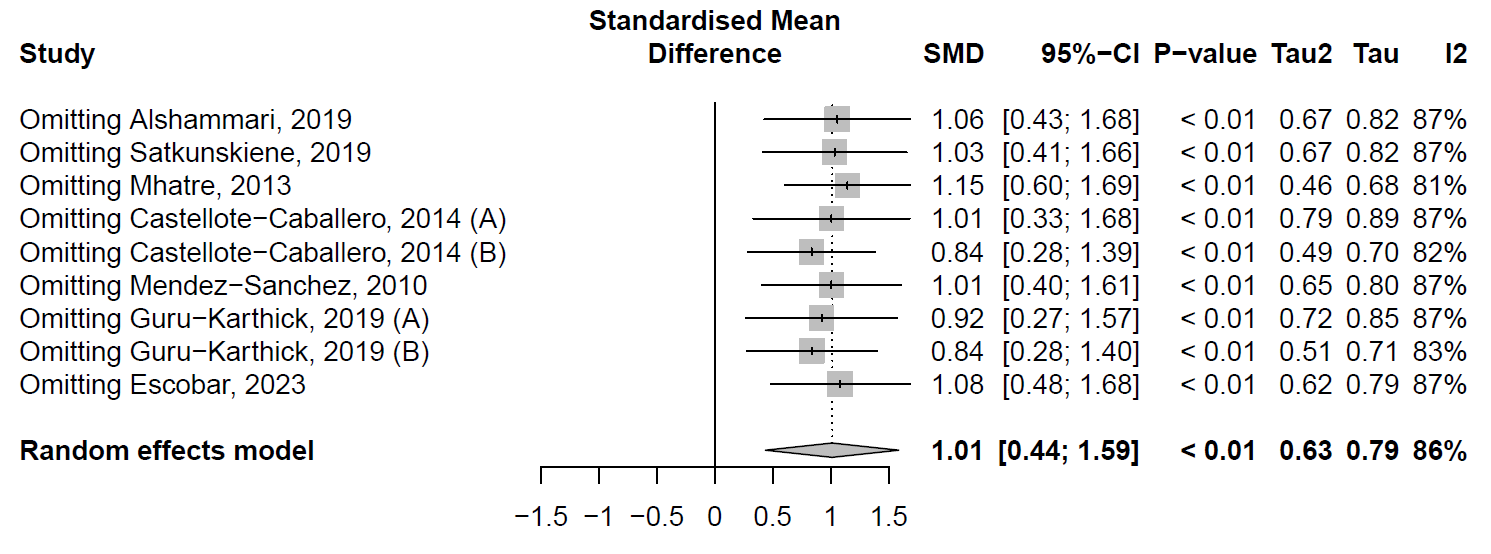


**S3 Figure.** Sensitivity analysis of short-term effects.


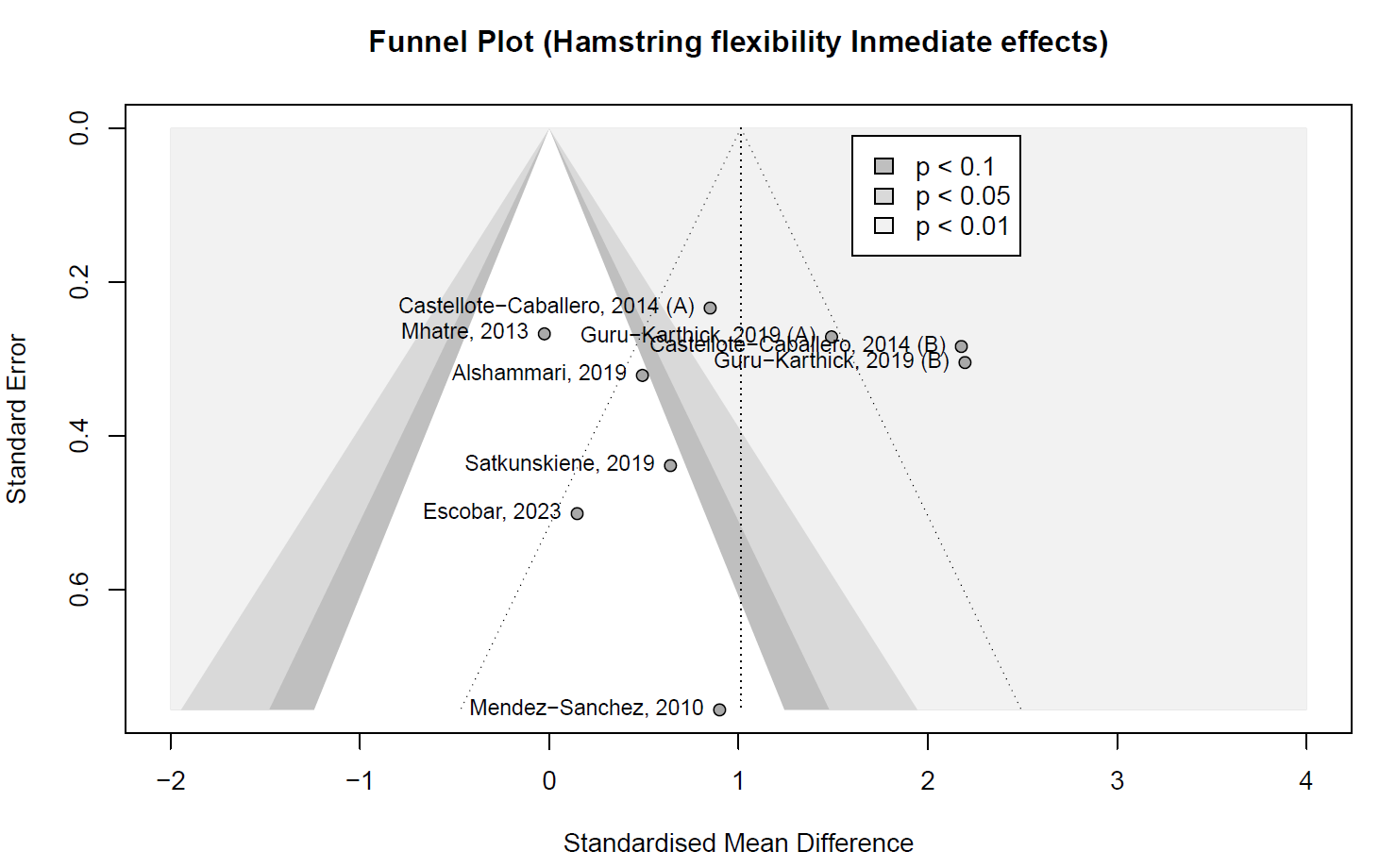


**S4 Figure.** Funnel plot of immediate effects.


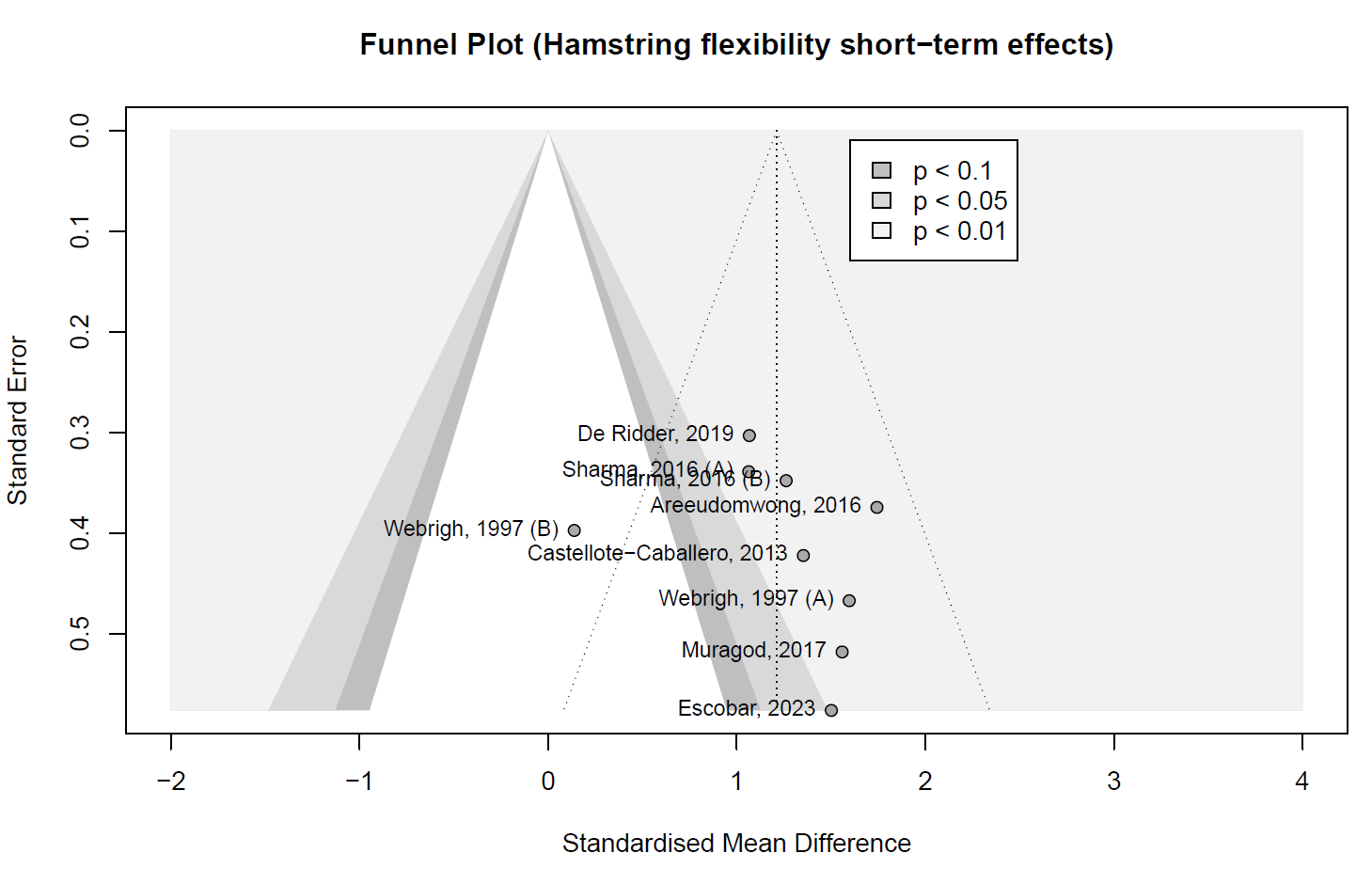


**S5 Figure**. Funnel plot of short-term effect.
